# Alcohol Consumption and Breast Cancer Risk among Women in Three Sub-Saharan African Countries

**DOI:** 10.1371/journal.pone.0106908

**Published:** 2014-09-08

**Authors:** Frank Qian, Temidayo Ogundiran, Ningqi Hou, Paul Ndom, Antony Gakwaya, Johashaphat Jombwe, Imran Morhason-Bello, Clement Adebamowo, Adeyinka Ademola, Oladosu Ojengbede, Olufunmilayo I. Olopade, Dezheng Huo

**Affiliations:** 1 Center for Clinical Cancer Genetics and Global Health, Department of Medicine, University of Chicago, Chicago, Illinois, United States of America; 2 Department of Surgery, College of Medicine, University of Ibadan, Ibadan, Nigeria; 3 Department of Health Studies, University of Chicago, Chicago, Illinois, United States of America; 4 Medical Oncology Service, Yaoundé General Hospital, Yaoundé, Cameroon; 5 Mulago Hospital, Makerere University, Kampala, Uganda; 6 Center for Population and Reproductive Health, University College Hospital, Ibadan, Nigeria; 7 Department of Epidemiology and Public Health, University of Maryland, Baltimore, Maryland, United States of America; The Institute of Cancer Research, United Kingdom

## Abstract

**Background:**

Alcohol drinking is linked to the development of breast cancer. However, there is little knowledge about the impact of alcohol consumption on breast cancer risk among African women.

**Methods:**

We conducted a case-control study among 2,138 women with invasive breast cancer and 2,589 controls in Nigeria, Cameroon, and Uganda from 1998 to 2013. A structured questionnaire was used to collect information on alcohol consumption, defined as consuming alcoholic beverages at least once a week for six months or more. Logistic regression was used to estimate adjusted odds ratio (aOR) and 95% confidence interval (CI).

**Results:**

Among healthy controls, the overall alcohol consumption prevalence was 10.4%, and the prevalence in Nigeria, Cameroon, and Uganda were 5.0%, 34.6%, and 50.0%, respectively. Cases were more likely to have consumed alcohol (aOR = 1.62, 95% CI: 1.33–1.97). Both past (aOR = 1.54; 95% CI: 1.19–2.00) and current drinking (aOR = 1.71; 95% CI: 1.30–2.23) were associated with breast cancer risk. A dose-response relationship was observed for duration of alcohol drinking (*P*-trend <0.001), with 10-year increase of drinking associated with a 54% increased risk (95% CI: 1.29–1.84).

**Conclusion:**

We found a positive relationship between alcohol consumption and breast cancer risk, suggesting that this modifiable risk factor should be addressed in breast cancer prevention programs in Africa.

## Introduction

Breast cancer is a leading cause of cancer morbidity and mortality for women world-wide [1], [2], [3], [4], [5], [6]. In recent decades, breast cancer incidence has seen a steady increase in many developing countries while it reached a plateau in developed nations [6], [7], [8]. Alcohol consumption has been considered a plausible risk factor for breast cancer. This relationship has been confirmed in some studies [9], [10], [11], [12], [13], [14], [15], but not in others [16], [17], [18], [19]. However, the majority of these studies were conducted among women in high income countries [9], [18], [20], [21], [22], with few in developing countries [19], [23], [24]. To our knowledge, there is no large study on alcohol intake and breast cancer conducted in Africa. Studies of the relationship between alcohol consumption and breast cancer risk among African Americans have found inconclusive results [16], [25], [26], [27], [28]. The extent of alcohol drinking's effect on breast cancer risk may vary across races, possibly due to different drinking habits, metabolism and genetic factors. In general, alcohol drinking is less common among African women than their counterparts in North America and Europe [29], but the prevalence rates vary by country in Africa: ever drinking prevalence ranges from <1% to over 40% and current drinking prevalence ranges from none to nearly 30% [29], [30], [31]. The general trend among African women is towards greater prevalence of alcohol consumption [29], [32].

Most studies of alcohol and breast cancer have assessed alcohol use in terms of ever-drinking versus nondrinking or the average daily dose [33]. Other patterns of alcohol use such as duration of alcohol consumption or age when first started drinking and their relationship to breast cancer risk are less well understood [12]. We conducted a case-control study of alcohol consumption patterns and breast cancer risk among women in three sub-Saharan African countries: Nigeria, Cameroon, and Uganda.

## Materials and Methods

### Study population

The African Breast Cancer Study was originally started in Nigeria in 1998 and was expanded to Uganda and Cameroon in 2011 using the same questionnaire and protocol. The study protocol was reviewed by the institutional review boards of the three sites and the University of Chicago (University of Chicago Biological Sciences Division Institutional Review Board (13304B and 10-023-B), University of Ibadan/University College Hospital Ethics Committee (UI/IRC/02/0003), Cameroon National Ethics Committee (N141/CNE/SE/2010), and Makerere University College of Health Sciences Ethics Committee (2011-023)). The study setting and design at the Nigeria site are described in detail elsewhere [34], [35], [36]. Briefly, cases were identified through University College Hospital (UCH), University of Ibadan, in Ibadan, Nigeria. Serving a population of more than 3 million people, UCH is a referral center for other hospitals and thus treat the majority of breast cancer cases in the region. All consecutive females who were 18 years or older, black of African descent, capable of providing informed consent, and had a histologic or clinical diagnosis of invasive breast cancer were recruited through the surgical oncology and radiotherapy units of the UCH. Controls were females aged 18 years or older, absent of breast cancer, and able to give informed consent. During the period of case enrollment, several communities were randomly selected by ballot from the list of all the communities in catchment area of UCH and considered to be stable, socio-economically and ethnically diverse, and represent UCH patients. Field interviewers randomly approached households in these communities and invited eligible women to visit community centers for the study. We also enrolled clinical controls through general outpatient and ophthalmology clinics in the UCH, matched for age and ethnicity. Women were enrolled during their waiting in clinics and they are unselected for their medical conditions. As the characteristics of hospital-based and community controls were similar, they were pooled in the further analysis. In Uganda, cases were identified at the breast and endocrine unit in department of surgery of the Mulago Hospital, Makerere University in Kampala. Mulago Hospital is a national referral hospital in Uganda and serves the 1.3 million residents in Kampala. Controls were randomly recruited from the general outpatient clinics and surgical ward admissions at Mulago Hospital, matched to cases for age and ethnicity. In Cameroon, breast cancer cases were enrolled at the department of medical oncology of Yaoundé General Hospital, Yaoundé University I in Yaoundé. Yaoundé General Hospital serves a population of 2.5 million people. Controls were randomly recruited from the clinics of general medicine and obstetrics and gynecology departments at the same hospital, matched to cases for age and ethnicity. At the Kampala and Yaoundé sites, hospital controls were unselected for their medical conditions. All study participants provided written informed consent prior to their interview. During the recruitment, both patients and controls were enthusiastic to participate in the study, with a response rate of >90%.

### Data collection and measures

Trained interviewers at the three sites performed structured questionnaire interviews, measured height and weight, and obtained blood samples. The questionnaire included questions about demographics, family history of breast cancer and history of benign breast disease, lifestyle factors, menstrual and reproductive history, and alcohol consumption patterns.

Before 2006, alcohol consumption was asked in Nigeria in terms of average volume of alcohol consumed per week (among 1,492 women). After 2006, the measurement was changed to average number of drinks per week. In Uganda and Cameroon, the alcohol consumption was asked in terms of average number of drinks per week. To convert the amount of alcohol into a common unit, the following conversion criteria were used: 350 mL bottle of beer (5% alcohol volume) ≈150 mL glass of wine (including local wine such as tonto or ajon in Uganda, 15% alcohol) ≈50 mL shot of spirits (including local spirits such as waragi or enguli in Uganda, 40% alcohol) ≈18 mL pure ethanol, which is equivalent to one standard drink. We then summed the amount of pure ethanol the participant consumed irrespective of the type of drink. We subsequently converted the volume of pure ethanol to gram: 1 mL  = 0.8 gram. Each standard drink is hence equivalent to about 14.4 grams of pure ethanol. We also constructed a new variable called the life-time alcohol consumption, which was defined as gram-years: the average daily amount of alcohol in grams was multiplied by the subject's duration of alcohol consumption in years.

We defined alcohol drinking as having ever consumed alcoholic beverages at least once a week for continuous six months or more. In addition, we measured other alcohol drinking patterns: status of drinking (never drank, past drank, and current drinking; current drinking is defined as consuming alcohol at least once a week up until one year before breast cancer diagnosis or interview date), age at first drink, duration of alcohol drinking, and average amount of alcohol consumed daily. Duration of alcohol drinking was grouped into four categories: never drank, 1–9, 10–19, and ≥20 years. Average amount of alcohol consumed daily was also grouped into four categories: never drank, 0.1–4.9, 5.0–9.9, and ≥10.0 grams. Age at first drink had five categories: never drank, ≤18, 19–24, 25–29, and ≥30 years. Life-time alcohol consumption was also categorized into five groups: never drank, 0.1–29.9, 30.0–79.9, 80.0–159.9, and ≥160.0 gram-years.

### Statistical analyses

Demographic factors and potential confounding factors for breast cancer were compared between cases and controls using student *t*-test or Wilcoxon rank-sum test for continuous variables and χ^2^ test or Fisher's exact test for categorical variables. All P-values reported are two-sided. We calculated the percentages of control women who were currently consuming alcohol by the age groups of 18–34, 35–44, 45–54, and ≥55 years.

Multiple logistic regression models were used to examine the association between breast cancer and various patterns of alcohol consumption while adjusting for study site and other potential confounders listed below. These confounders were adjusted for in every multivariable logistic regression model and were defined as follows: age at diagnosis or interview (5-year interval categories), ethnicity (Yoruba, Ibo, Hausa, Baganda, Bantous, Semi-Bantous, others), education (none, elementary, secondary, some college or above, and vocational), age at menarche (≤14, 15–16, 17–18, ≥19 year), family history of breast cancer (yes, no), history of benign breast disease (yes, no), hormonal contraceptive use (ever, never), fertility drugs (ever, never), height, body mass index (BMI, kg/m^2^), menopausal status (premenopausal, natural postmenopausal, artificial postmenopausal), age at first live birth (<20, 20–24, 25–29, ≥30 years), and parity (0, 1–2, 3–4, 5–6, ≥7). Adjusted odds ratio (aOR) and 95% confidence interval (CI) were computed from the logistic regression models. Ever drank, status of drinking, and type of alcohol consumed (never drank, beer, wine, spirits) were included in the models solely as categorical variables. Age at first drink, duration of alcohol drinking, average amount of alcohol consumed daily, and life-time alcohol consumption were analyzed as both categorical variables and continuous variables. Stratified analyses were also performed to compare the breast cancer risk between ever-drinkers vs. never-drinkers across different strata of demographic and reproductive variables, such as premenopausal vs. post-menopausal women. Potential confounding variables were adjusted for in these analyses.

There were missing data for age at first drink, duration of alcohol drinking, average volume of drinking, and other important covariates. To use all available information and avoid bias due to list-wise deletion in the multivariable analysis, we conducted multiple imputations via chained equations approach for imputation [37], [38]. All covariates and outcome variable (case-control status) were included in the imputation model. We ran 100 imputations to improve statistical efficiency of the imputation estimates to be over 99.5%. The logistic regression models described above were fitted using each of the imputed data sets. Results across the multiply imputed datasets were combined according to Rubin's rules [39]. Multiple imputation approach assumes missing at random. Older women tended to forget how much alcohol they consumed in the past. By including age in the multiple imputation models, it is reasonable to believe that the probability of missing alcohol data was at random. As a sensitivity analysis, we conducted the analysis using data without missing value in any of the covariates in the models (i.e. complete case analysis). All statistical analyses, including the imputation (*ice* module), were conducted with STATA 13.0 (StataCorp, College Station, TX, USA).

## Results

A total of 2,138 breast cancer cases and 2,589 controls (women without breast cancer) were enrolled in the study between March 1998 and July 2013, including 1,756 cases and 2,217 controls in Nigeria, 192 cases and 182 controls in Cameroon, and 190 cases and 190 controls in Uganda. Table 1 shows selected demographic and reproductive characteristics of study participants. Since Nigeria comprises the largest proportion of the study population, demographic differences between cases and controls in the Nigerian population will influence the difference in the overall population. Cases in Nigeria were 5.1 years older than controls on average; there was no difference in age between cases and controls in Cameroon or Uganda. Most participants in Nigeria were Yoruba, and over half of the participants in Uganda were Baganda and in Cameroon were Bantous. In all study participants, about 40% had less than a secondary education. About 75% of women had ≥3 live births. There were no differences between cases and controls in terms of their average age at menarche and use of fertility drugs in the crude analysis. In the Nigerian population, cases were taller than controls, on average. Cases were more likely to have a family history of breast cancer and a history of benign breast disease in Nigeria and Cameroon. Similarly, controls were more likely to have ever used hormonal contraceptives. In the Cameroon population, the proportion of cases who had a family history of breast cancer (23.4%) was considerably larger than the controls (8.2%). This difference was less pronounced in Nigeria and Uganda, though the difference was still statistically significant in Nigeria.

**Table 1:** Demographics of women with invasive breast cancer (cases) and those without (controls) in Nigeria, Cameroon and Uganda, 1998–2013.

Demographics	Nigeria	Cameroon		Uganda			Total	
	Case (N = 1756)	Control^a^ (N = 2217)	Case (N = 192)	Control^a^ (N = 182)	Case (N = 190)	Control^a^ (N = 190)	Case (N = 2138)	Control^a^ (N = 2589)
**Age, year (mean ± SD)**	47.6±11.7	42.5±13.0	47.6±11.4	47.5±11.7	46.6±12.7	46.3±12.2	47.5±11.8	43.1±13.0
** ** ***P*** **-value**		<0.001		0.94		0.80		<0.001
**Ethnicity, n (%)**								
** Yoruba**	1,288 (73.4)	1,816 (81.2)					1,288 (60.2)	1,816 (68.3)
** Ibo**	226 (12.9)	101 (5.0)					226 (10.6)	101 (4.2)
** Hausa**	22 (1.3)	171 (8.0)					22 (1.0)	171 (6.8)
** Baganda**					83 (43.7)	84 (44.2)	83 (3.9)	84 (3.4)
** Bantous**			101 (52.6)	94 (53.3)			101 (4.7)	94 (4.4)
** Semi-Bantous**			74 (38.5)	85 (45.1)			74 (3.5)	85 (4.3)
** Other (Cameroon)**			17 (8.9)	3 (1.5)			17 (0.8)	3 (0.1)
** Other (Nigeria)**	220 (12.5)	129 (5.8)					220 (10.3)	129 (4.9)
** Other (Uganda)**					107 (56.3)	106 (55.8)	107 (5.0)	106 (4.4)
** ** ***P*** **-value**		<0.001		0.02		0.99		<0.001
**Education, n (%)**								
** No formal**	354 (20.2)	315 (14.0)	8 (4.2)	2 (0.7)	21 (11.1)	11 (6.9)	383 (17.9)	328 (12.3)
** Elementary**	415 (23.6)	481 (22.2)	58 (30.2)	46 (22.8)	76 (40.0)	90 (46.3)	549 (25.7)	617 (24.4)
** Secondary**	354 (20.2)	534 (22.8)	84 (43.8)	101 (57.9)	59 (31.1)	46 (22.5)	497 (23.3)	681 (25.7)
** Some college or above**	418 (23.8)	607 (28.3)	33 (17.2)	23 (12.3)	18 (9.5)	15 (6.4)	469 (21.9)	645 (25.2)
** Vocational/technical**	215 (12.2)	280 (12.6)	9 (4.7)	10 (6.4)	16 (8.4)	28 (17.9)	240 (11.2)	318 (12.5)
** ** ***P*** **-value**		0.30		0.14		0.16		0.21
**Family history of breast cancer, n (%)**								
** Yes**	107 (6.1)	77 (3.3)	45 (23.4)	15 (8.5)	7 (3.7)	10 (4.2)	159 (7.4)	102 (3.8)
** No**	1,649 (93.9)	2,140 (96.7)	147 (76.6)	167 (91.5)	183 (96.3)	180 (95.8)	1,979 (92.6)	2,487 (96.2)
** ** ***P*** **-value**		<0.001		<0.001		0.68		<0.001
**Benign breast disease, n (%)**								
** Yes**	173 (9.9)	149 (7.4)	20 (10.4)	9 (5.3)	16 (8.4)	8 (2.7)	209 (9.8)	166 (6.9)
** No**	1,583 (90.1)	2,068 (92.6)	172 (89.6)	173 (94.7)	174 (91.6)	182 (97.3)	1,929 (90.2)	2,423 (93.1)
** ** ***P*** **-value**		0.004		0.04		0.01		<0.001
**Menopause status**								
** Premenopausal**	925 (52.7)	1,545 (69.0)	131 (68.2)	123 (71.6)	110 (57.9)	114 (62.9)	1,166 (54.5)	1,782 (68.7)
** Postmenopausal, artificial**	94 (5.3)	64 (3.2)	19 (9.9)	8 (4.5)	11 (5.8)	5 (3.1)	124 (5.8)	77 (3.3)
** Postmenopausal, natural**	737 (42.0)	608 (27.8)	42 (21.9)	51 (23.9)	69 (36.3)	71 (33.9)	848 (39.7)	730 (28.0)
** ** ***P*** **-value**		<0.001		0.06		0.34		<0.001
**Age at natural menopause (mean ± SD)**	48.8±5.3	49.0±5.1	48.1±4.4	48.4±4.5	47.5±4.6	48.3±5.6	48.7±5.2	48.9±5.1
** ** ***P*** **-value**		0.54		0.70		0.09		0.31
**Hormonal contraceptives, n (%)**								
** Yes**	495 (28.2)	706 (35.0)	51 (26.6)	77 (43.4)	83 (43.7)	88 (45.3)	629 (29.4)	871 (36.6)
** No**	1,261 (71.8)	1,511 (65.0)	141 (73.4)	105 (56.6)	107 (56.3)	102 (54.8)	1,509 (70.6)	1,718 (63.4)
** ** ***P*** **-value**		0.002		0.001		0.84		<0.001
**Fertility drugs, n (%)**								
** Yes**	102 (10.1)	137 (11.3)	28 (14.7)	29 (17.7)	7 (3.8)	1 (0.9)	137 (9.9)	167 (10.8)
** No**	909 (89.9)	1,295 (88.7)	162 (85.3)	153 (82.3)	177 (96.2)	188 (99.1)	1,248 (90.1)	1,636 (89.2)
** ** ***P*** **-value**		0.59		0.71		0.13		0.83
**Age at menarche, year (mean ± SD)**	15.3±2.1	15.3±2.2	14.2±1.6	14.2±1.7	14.7±1.6	14.7±1.6	15.1±2.0	15.1±2.1
** ** ***P*** **-value**		0.23		0.97		0.75		0.16
**Height, cm (mean ± SD)**	161.1±7.7	159.0±6.5	164.4±6.2	163.9±6.4	161.0±8.5	162.5±9.9	161.4±7.7	159.6±6.9
** ** ***P*** **-value**		<0.001		0.27		0.13		<0.001
**BMI, kg/m^2^ (mean ± SD)**	25.8±6.0	27.0±5.7	27.1±5.4	28.2±4.5	24.2±4.9	25.8±5.1	25.8±5.9	27.0±5.6
** ** ***P*** **-value**		<0.001		0.07		0.03		<0.001
**Radiation exposure, n (%)**								
** Yes**	11 (1.1)	187 (13.8)	46 (24.0)	5 (2.7)	20 (10.5)	15 (6.7)	77 (5.5)	207 (11.9)
** No**	1,006 (98.9)	1,252 (86.2)	146 (76.0)	177 (97.3)	170 (89.5)	175 (93.3)	1,322 (94.5)	1,604 (88.1)
** ** ***P*** **-value**		<0.001		<0.001		0.37		<0.001
**Parity, n (%)**								
** 0**	159 (9.1)	254 (5.0)	21 (10.9)	15 (7.5)	29 (15.3)	11 (5.0)	209 (9.8)	280 (5.3)
** 1**–**2**	308 (17.5)	474 (17.7)	45 (23.4)	52 (28.3)	40 (21.1)	47 (22.3)	393 (18.4)	573 (18.7)
** 3**–**4**	543 (30.9)	708 (37.1)	60 (31.3)	44 (26.6)	33 (17.4)	47 (26.5)	636 (29.8)	799 (35.4)
** 5**–**6**	498 (28.4)	524 (27.6)	32 (16.7)	41 (23.8)	49 (25.8)	40 (23.9)	579 (27.1)	605 (27.0)
** ≥7**	248 (14.1)	257 (12.6)	34 (17.7)	30 (13.8)	39 (20.5)	45 (22.2)	321 (15.0)	332 (13.7)
** ** ***P*** **-value**		<0.001		0.24		0.01		<0.001
**Age at first live birth, year (mean ± SD)^b^**	23.3±4.9	23.4±4.8	21.3±4.9	21.5±5.6	20.4±4.4	20.8±4.7	22.9±4.9	23.0±4.9
** ** ***P*** **-value**		0.77		0.62		0.56		0.92
**Ever breast feed, n (%)**								
** Yes**	1,581 (90.8)	1,950 (94.9)	167 (87.0)	160 (88.6)	154 (82.4)	177 (95.6)	1,902 (89.7)	2,287 (94.4)
** No**	161 (9.2)	254 (5.1)	25 (13.0)	22 (11.4)	33 (17.7)	11 (4.4)	219 (10.3)	287 (5.6)
** ** ***P*** **-value**		<0.001		0.84		<0.001		<0.001
**Lifetime duration of breastfeeding, month, n (%)**								
** ≤24**	417 (23.9)	599 (18.2)	68 (35.4)	71 (37.9)	62 (33.2)	46 (22.6)	547 (25.8)	716 (20.1)
** 25**–**48**	387 (22.2)	476 (23.2)	46 (24.0)	36 (21.9)	24 (12.8)	25 (12.1)	457 (21.6)	537 (22.2)
** 49**–**72**	374 (21.5)	492 (26.6)	36 (18.8)	32 (18.9)	26 (13.9)	36 (20.1)	436 (20.6)	560 (25.5)
** 73**–**96**	241 (13.8)	264 (13.6)	22 (11.5)	14 (7.8)	19 (10.2)	21 (12.1)	282 (13.3)	299 (13.0)
** >96**	323 (18.5)	373 (18.4)	20 (10.4)	29 (13.6)	56 (30.0)	60 (33.2)	399 (18.8)	462 (19.2)
** ** ***P*** **-value**		<0.001		0.30		0.42		<0.001

**Note**: Numbers in parentheses are proportions (%). SD: standard deviation.

a Proportions or means for the controls are adjusted values based on the age distribution of cases; *P*-values were also age-adjusted in logistic regressions.

b Among parous women.

Overall, there were 1,311 (1.7%) missing values across 16 variables in Tables 1 and 2 (excluding derivative variables). Complete information was available for ethnicity, ever drink alcohol, menopausal status, and site of study. About 5% of participants had a missing value for age at menarche, and 3% of them had missing values for height and weight. Data were occasionally missing for other variables as well. One hundred data sets were generated by multiple imputation such that the efficiency of estimating OR was greater than 99.5% for the fraction of missing information up to 0.08.

**Table 2:** Patterns of alcohol consumption among women in Nigeria, Cameroon and Uganda, 1998–2013

Variable	Nigeria		Cameroon		Uganda		Total	
	No. of Cases, n (%)	No. of Controls, n (%)	No. of Cases, n (%)	No. of Controls, n (%)	No. of Cases, n (%)	No. of Controls, n (%)	No. of Cases, n (%)	No. of Controls, n (%)
**Ever drank alcohol**								
** No**	1,546 (88.0)	2,106 (95.0)	94 (49.0)	119 (65.4)	90 (47.4)	95 (50.0)	1,730 (80.9)	2,320 (89.6)
** Yes**	210 (12.0)	111 (5.0)	98 (51.0)	63 (34.6)	100 (52.6)	95 (50.0)	408 (19.1)	269 (10.4)
**Status of drinking**								
** Never**	1,546 (89.3)	2,106 (95.4)	94 (49.2)	119 (65.8)	90 (48.1)	95 (51.1)	1,730 (82.0)	2,320 (90.1)
** Past**	117 (6.8)	55 (2.5)	17 (8.9)	19 (10.5)	59 (31.6)	51 (27.4)	193 (9.2)	125 (4.9)
** Current**	68 (3.9)	46 (2.1)	80 (41.9)	43 (23.8)	38 (20.3)	40 (21.5)	186 (8.8)	129 (5.0)
**Age at first drink, year**								
** Never**	1,546 (88.8)	2,106 (95.2)	94 (49.0)	119 (65.8)	90 (50.0)	95 (51.4)	1,730 (81.9)	2,320 (90.0)
** drank**								
** ≤18**	26 (1.5)	13 (0.6)	16 (8.3)	14 (7.7)	25 (13.9)	23 (12.4)	67 (5.2)	50 (1.9)
** 19**–**24**	64 (3.7)	32 (1.5)	36 (18.8)	22 (12.2)	26 (14.4)	26 (14.1)	126 (6.0)	80 (3.1)
** 25**–**29**	42 (2.4)	28 (1.3)	27 (14.1)	8 (4.4)	12 (6.7)	13 (7.0)	81 (3.8)	49 (1.9)
** ≥30**	63 (3.6)	34 (1.5)	19 (9.9)	18 (9.9)	27 (15.0)	28 (15.1)	109 (5.2)	80 (3.1)
**Mean ± SD**	26.7±9.3	26.3±7.8	24.0±6.6	23.9±8.2	24.3±8.5	24.0±9.4	25.5±8.6	24.9±8.6
***P*** **-value**		0.72		0.93		0.85		0.45
**Duration of alcohol drinking, year**								
** Never drank**	1,546 (89.0)	2,106 (95.1)	94 (50.3)	119 (73.0)	90 (55.6)	95 (62.1)	1,730 (82.9)	2,320 (91.7)
** 1**–**9**	63 (3.6)	59 (2.7)	17 (9.2)	9 (5.5)	30 (18.5)	24 (15.7)	110 (5.3)	92 (3.6)
** 10**–**19**	69 (4.0)	32 (1.5)	27 (14.6)	12 (7.4)	18 (11.1)	13 (8.5)	114 (5.5)	57 (2.3)
** ≥ 20**	60 (3.5)	17 (0.8)	48 (26.0)	23 (14.1)	24 (14.8)	21 (13.7)	132 (6.3)	61 (2.4)
**Mean ± SD** †	15.0±10.3	10.3±9.1	21.1±11.8	20.0±12.7	17.2±12.8	14.8±12.6	17.0±11.6	14.2±11.9
***P*** **-value**		<0.001		0.57		0.18		0.002
**Average amount of alcohol consumed daily, gram**								
** Never**	1,546 (90.5)	2,106 (96.2)	94 (49.2)	119 (65.8)	90 (71.4)	95 (69.9)	1,730 (85.4)	2,320 (92.5)
** Drank**	51 (3.0)	18 (0.8)	42 (22.0)	19 (10.5)	20 (15.9)	24 (17.7)	113 (5.6)	61 (2.4)
** 5.0**–**9.9**	66 (3.9)	35 (1.6)	28 (14.7)	22 (12.2)	7 (5.6)	13 (9.6)	101 (5.0)	70 (2.8)
** ≥10**	46 (2.7)	31 (1.4)	27 (14.1)	21 (11.6)	9 (7.1)	4 (2.9)	82 (4.1)	56 (2.2)
**Mean ± SD** †	10.1±28.7	13.2±39.4	11.9±29.9	8.8±7.6	10.1±15.6	9.3±13.0	10.6±26.4	10.8±26.7
***P*** **-value**		0.43		0.42		0.69		0.92
**Life-time alcohol consumption, gram-years**								
** Never**	1,546 (90.9)	2,106 (96.2)	94 (50.3)	119 (73.0)	90 (70.9)	95 (73.1)	1,730 (86.0)	2,320 (93.3)
** Drank**	43 (2.4)	36 (1.6)	12 (5.5)	5 (3.1)	6 (5.1)	9 (6.9)	56 (2.8)	52 (2.0)
** 30.0**–**79.9**	47 (2.8)	20 (0.8)	29 (13.7)	9 (5.5)	6 (5.1)	11 (8.5)	78 (3.9)	39 (1.5)
** 80.0**–**159.9**	42 (2.1)	12 (0.7)	17 (12.6)	11 (6.7)	9 (7.7)	10 (7.7)	68 (3.4)	32 (1.5)
** ≥160.0**	23 (1.9)	15 (0.7)	33 (18.0)	19 (11.7)	13 (11.1)	5 (3.9)	78 (3.9)	39 (1.7)
**Mean ± SD** †	144.0±370.1	238.1±888.5	319.1±1211.4	186.2±182.0	179.0±211.6	84.3±66.9	205.1±748.8	190.8±644.6
***P*** **-value**		0.25		0.47		0.01		0.84
**Type of alcohol** 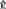								
** Never drank**	1,546 (88.0)	2,106 (95.0)	94 (49.0)	119 (65.4)	90 (47.4)	95 (50.0)	1,730 (80.9)	2,320 (89.6)
** Beer**	166 (9.5)	93 (4.2)	84 (43.8)	61 (33.5)	20 (10.5)	28 (14.7)	270 (12.6)	182 (7.0)
** Wine**	35 (2.0)	16 (0.7)	29 (15.1)	25 (13.7)	19 (10.0)	16 (8.4)	83 (3.9)	57 (2.2)
** Spirits**	27 (1.5)	16 (0.7)	22 (11.5)	15 (8.2)	17 (9.0)	13 (6.8)	66 (3.1)	44 (1.7)

† Among drinkers.

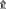
Includes drinkers who drink a combination of alcoholic beverages, percentages may not add up to 100%.

**Figure 1:**
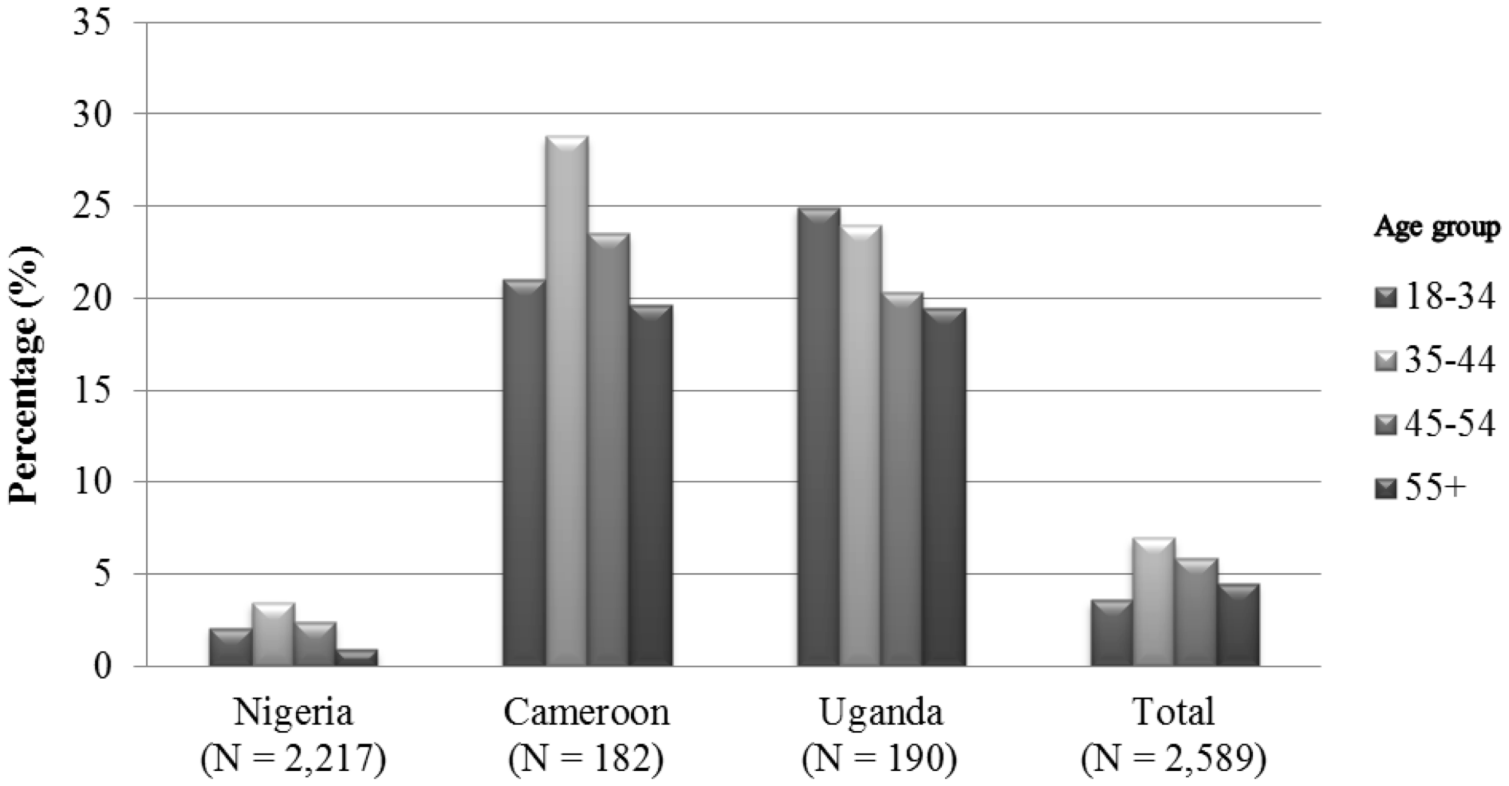
Percentage of healthy controls who were current drinkers by age group in the three African countries.

Among healthy controls, 10.4% were alcohol drinkers, and the percentage of ever drinkers in Cameroon, Nigeria, and Uganda were 34.6%, 5.0%, and 50.0%, respectively. Figure 1 shows the percentages of current alcohol drinking among women by age group. Women in Cameroon and Uganda were more likely to be current drinkers than those in Nigeria. Among women in Cameroon and Nigeria, current drinking was proportionately more common in middle-aged women (aged 35–44 and 45–54 years) than younger or older women, while in Uganda, young women (aged 18–34) had the highest prevalence of current drinking.

Table 2 presents the alcohol drinking patterns among cases and controls. There was no statistical difference in the mean age at first drink and mean amount of alcohol consumed daily in the overall data. In terms of duration of alcohol consumption, cases were found to drink for 2.8 years longer on average than in controls. In the overall population, 86.4% of drinkers consumed ≤1 drink per day (1 drink  = 14.4 g of pure ethanol), 8.8% of drinkers consumed 1–2 drinks per day, 2.8% of drinkers consumed 2–3 drinks per day, and 2.1% of drinkers consumed more than 3 drinks per day. In drinkers, there was no considerable difference in amount of alcohol drinking among the three countries.

Table 3 presents the relationship of the alcohol drinking patterns to breast cancer risk with calculated odds ratio and 95% CI using imputed data. Among the whole study sample, cases were more likely than controls to have ever consumed alcohol (aOR: 1.62, 95% CI: 1.33–1.97). Both past drinking (aOR: 1.54; 95% CI: 1.19–2.00) or current drinking (aOR: 1.71; 95% CI: 1.30–2.23) were associated with increased breast cancer risk. Breast cancer risk was relatively consistent regardless of when the women first started drinking. Compared with non-drinkers, women who had drunk for <10 years had 41% higher odds of having breast cancer (aOR: 1.41; 95% CI: 1.04–1.91), and those who had drunk for 10–19 years had 71% (aOR: 1.71; 95% CI: 1.23–2.39) and those for ≥20 years had 82% (aOR: 1.82; 95% CI: 1.30–2.54) higher odds of having breast cancer. Every 10-year increase in the duration of drinking was associated with a 54% increase in breast cancer risk (aOR: 1.54; 95% CI: 1.29–1.84; *P*-trend: <0.001). We also found that alcohol consumption was associated with breast cancer risk, regardless of alcohol amount; breast cancer risk increase was shown even in the category of women who consume 0.1–4.9 g/day (aOR: 1.82, 95% CI: 1.31–2.54). In the variable life-time alcohol consumption, increased breast cancer risk was observed for subjects who consume at least 30 gram-years or more of alcohol (aOR: 1.85, 95% CI: 1.26–2.72). We also found a statistically significant trend, with each 100 gram-years contributing a 29% increase in breast cancer risk (aOR: 1.29; 95% CI: 1.12–1.51; *P*-trend  = 0.001). The complete case analysis, in which subjects with missing values in any of the variables in the models were dropped, showed similar study findings (Table S1).

**Table 3:** Multivariable logistic regression analyses of alcohol consumption and breast cancer risk among women in Nigeria, Cameroon and Uganda, 1998–2013, using data after multiple imputation (N = 4727).

Variable	Nigeria	Cameroon	Uganda	Overall
	Adjusted OR	Adjusted OR	Adjusted OR	Adjusted OR
	(95% CI)†	(95% CI)†	(95% CI)†	(95% CI)‡
**Ever drank alcohol**				
** No**	1.0 (ref.)	1.0 (ref.)	1.0 (ref.)	1.0 (ref.)
** Yes**	1.80 (1.38–2.35)	1.80 (1.11–2.92)	1.00 (0.62–1.62)	1.62 (1.33–1.97)
**Status of drinking**				
** Never**	1.0 (ref.)	1.0 (ref.)	1.0 (ref.)	1.0 (ref.)
** Past**	1.88 (1.33–2.67)	1.00 (0.45–2.23)	0.99 (0.57–1.75)	1.54 (1.19–2.00)
** Current**	1.70 (1.13–2.55)	2.17 (1.28–3.69)	1.01 (0.55–1.85)	1.71 (1.30–2.23)
**Age at first drink, year**				
** Never drank**	1.0 (ref.)	1.0 (ref.)	1.0 (ref.)	1.0 (ref.)
** ≤18**	2.34 (1.14–4.80)	1.21 (0.49–2.96)	1.09 (0.53–2.23)	1.68 (1.11–2.54)
** 19**–**24**	1.94 (1.20–3.14)	1.59 (0.80–3.16)	0.95 (0.46–1.93)	1.74 (1.26–2.41)
** 25**–**29**	1.53 (0.91–2.56)	4.09 (1.63–10.2)	0.81 (0.31–2.11)	1.77 (1.20–2.61)
** ≥30**	1.72 (1.10–2.69)	1.47 (0.67–3.21)	1.09 (0.53–2.24)	1.39 (1.01–1.91)
***P*** **-value for trend**	<0.001	0.08	0.99	<0.001
**Duration of alcohol drinking, year**				
** Never**	1.0 (ref.)	1.0 (ref.)	1.0 (ref.)	1.0 (ref.)
** Drank**	1.35 (0.92–1.99)	1.25 (0.51 3.05)	0.94 (0.49–1.80)	1.41 (1.04–1.91)
** 10**–**19**	1.90 (1.22–2.97)	1.63 (0.75–2.74)	1.16 (0.52–2.57)	1.71 (1.23–2.39)
** ≥ 20**	2.97 (1.66–5.31)	2.30 (1.19–4.45)	0.99 (0.49–2.00)	1.82 (1.30–2.54)
***P*** **-value for trend**	<0.001	0.002	0.85	<0.001
**Per 10-year increase**	1.84 (1.43–2.37)	1.85 (1.24–2.75)	1.04 (0.68–1.60)	1.54 (1.29–1.84)
**Average amount of alcohol** **consumed daily, gram**				
** Never drank**	1.0 (ref.)	1.0 (ref.)	1.0 (ref.)	1.0 (ref.)
** 0.1**–**4.9**	2.27 (1.33–3.87)	2.62 (1.31–5.20)	0.99 (0.53–1.87)	1.82 (1.31–2.54)
** 5.0**–**9.9**	1.91 (1.23–2.96)	1.31 (0.65–2.64)	0.72 (0.31–1.69)	1.52 (1.10–2.11)
** ≥10**	1.37 (0.85–2.22)	1.61 (0.77–3.35)	1.48 (0.58–3.80)	1.50 (1.04–2.18)
***P*** **-value for trend**	0.03	0.13	0.57	0.007
**Per 10g increase**	1.43 (1.05–1.96)	1.50 (0.89–2.52)	1.20 (0.63–2.28)	1.39 (1.09–1.76)
**Life-time alcohol consumption, gram-years**				
** Never**	1.0 (ref.)	1.0 (ref.)	1.0 (ref.)	1.0 (ref.)
** Drank**	1.24 (0.79–1.94)	1.24 (0.38–4.04)	0.90 (0.44–1.83)	1.29 (0.91–1.83)
** 30.0**–**79.9**	2.61 (1.49–4.58)	2.21 (0.96–5.11)	0.76 (0.32–1.82)	1.85 (1.26–2.72)
** 80.0**–**159.9**	2.11 (1.13–3.93)	1.96 (0.88–4.41)	0.81 (0.33–1.98)	1.73 (1.15–2.60)
** ≥160.0**	1.87 (1.01–3.45)	1.67 (0.83–3.36)	1.98 (0.75–5.26)	1.76 (1.17–2.64)
***P*** **-value for trend**	0.001	0.06	0.19	0.001
**Per 100 gram-year increase**	1.45 (1.16–1.81)	1.29 (0.99–1.70)	1.26 (0.89–1.77)	1.29 (1.12–1.51)
**Type of alcohol** 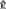				
** Never drank**	1.0 (ref.)	1.0 (ref.)	1.0 (ref.)	1.0 (ref.)
** Beer**	1.80 (1.35–2.40)	1.51 (0.92–2.46)	0.88 (0.48–1.61)	1.53 (1.22–1.91)
** Wine**	2.32 (1.20–4.50)	1.01 (0.52–1.98)	1.11 (0.58–2.13)	1.32 (0.91–1.92)
** Spirits**	1.76 (0.89–3.48)	1.31 (0.58–2.95)	1.30 (0.64–2.67)	1.52 (0.99–2.32)

† Adjusted for age at diagnosis or interview (categorical), ethnicity, education (categorical), age at menarche (categorical), number of live births (categorical), age at first live birth (categorical), menopausal status, family history of breast cancer, benign breast disease, hormonal contraceptive use, BMI (continuous), and height (continuous);

‡ Adjusted for the above variables and study site.

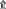
Includes drinkers who drink a combination of alcoholic beverages.

Table 4 shows the relationship between ever-drinking and breast cancer risk by strata of age at interview or diagnosis, ethnicity, height and other demographic and reproductive variables. When the interactions between the stratified variables and ever drinking alcohol were tested, parity and BMI were found to have a statistically significant interaction with alcohol drinking (*P* = 0.03 for both tests). In parous women, the association between drinking alcohol and breast cancer was weaker (aOR: 1.55; 95% CI: 1.26–1.91) than that in nulliparous women (aOR: 2.81; 95% CI: 1.24–6.35). There was no statistically significant association between alcohol consumption and breast cancer among women with BMI ≤25 kg/m^2^ (aOR: 1.09; 95% CI: 0.79–1.50), while among women with BMI >25 kg/m^2^, ever-drinkers had nearly twice the odds for breast cancer than never-drinkers (aOR: 2.06, 95% CI: 1.59–2.68). For other stratification variables, drinkers had similarly elevated risks for breast cancer compared to non-drinkers across the strata of these variables.

**Table 4:** Stratified analyses of alcohol consumption and breast cancer risk among women in Nigeria, Cameroon, and Uganda using data after multiple imputation, 1998–2013.

Stratified variable	Number of participants	Number and prevalence of ever-drinkers, n (%)	Adjusted OR for being ever-drinkers vs. non-drinkers (95% CI)*	*P*-value for interaction
**Age at interview or diagnosis, year**				
** <50**	3,047	384 (12.6)	1.64 (1.28–2.11)	
** ≥50**	1,680	293 (17.4)	1.97 (1.42–2.73)	0.49
**Menopausal status**				
** Premenopausal**	2,948	376 (12.8)	1.64 (1.26–2.12)	
** Post-menopausal**	1,779	301 (16.9)	1.82 (1.32–2.52)	0.14
**Parity**				
** Nulliparous**	489	68 (13.9)	2.81 (1.24–6.35)	
** Parous**	4,238	609 (14.4)	1.55 (1.26–1.91)	0.03
**Family history of breast cancer**				
** Yes**	261	85 (32.6)	2.66 (1.08–6.57)	
** No**	4,466	592 (13.3)	1.59 (1.29–1.96)	0.67
**Benign breast disease**				
** Yes**	375	86 (22.9)	2.43 (1.25–4.75)	
** No**	4,352	591 (13.6)	1.56 (1.26–1.93)	0.11
**Ever use of hormonal contraceptives**				
** Yes**	1,500	294 (19.6)	1.48 (1.09–2.02)	
** No**	3,227	383 (11.9)	1.73 (1.32–2.27)	0.58
**Body mass index, kg/m^2^**				
** ≤25**	2,209	292 (13.2)	1.09 (0.79–1.50)	
** >25**	2,518	385 (15.3)	2.06 (1.59–2.68)	0.03
**Height, cm**				
** ≤160**	2,510	286 (11.4)	1.64 (1.23–2.20)	
** >160**	2,217	391 (17.6)	1.72 (1.29–2.29)	0.11
**Age at menarche, year**				
** ≤15**	2,943	442 (15.0)	1.71 (1.33–2.20)	
** >15**	1,784	235 (13.2)	1.55 (1.09–2.21)	0.86
**Ethnicity**				
** Yoruba**	3,104	217 (7.0)	1.51 (1.12–2.04)	
** Other**	1,623	460 (28.3)	1.62 (1.27–2.07)	0.81

*Adjusted for all other variables listed in this table as well as age at first live birth and study site.

## Discussion

Our case-control study of 4,727 African women from Nigeria, Cameroon, and Uganda showed a positive relationship between breast cancer and alcohol drinking. Overall, the odds of having breast cancer were 62% higher among women who ever consumed alcohol than among no-drinkers. This finding was in general agreement with literature. The current evidence on the relationship between alcohol consumption and female breast cancer risk is largely based on studies conducted in Caucasian populations [40], [41], and the observed evidence in African American women was inconsistent [17], [18], [42], [43]. Numerous reasons may explain for the racial difference, including drinking habits, metabolism and genetic factors. For example, there are racial differences in distributions of genetic polymorphisms related to ethanol metabolism [44], [45]. Traditionally, alcohol consumption among African women is less common compared with those in developed countries. However, as women in Africa are increasingly influenced by western cultures and begin to change their lifestyle and as the populations in African countries are becoming more affluent, more and more women may be exposed to alcohol [29], [46], [47]. Few studies have investigated this relationship among African women [1]. Our study is the first multi-country study using a large African women sample. The incidence of breast cancer is anticipated to increase in African countries such as in Nigeria [48].

Our study also found that women who were past drinkers (54% increase) had almost the same elevated risk as women who were current drinkers (71% increase). Our study showed a dose-response relationship between duration of alcohol drinking and breast cancer risk; women who drank for 20 years or longer had almost twice the odds of being cancer cases compared with non-drinkers. This relationship was confirmed in some studies [12], [49] but not in others [12], [43]. We did not find dose-response relationship between amount of alcohol intake and breast cancer risk, possibly because there were few heavy drinkers in our study population. Studies have suggested that a moderate level of alcohol consumption (e.g., 5–10 g/day) may be sufficient for increasing breast cancer risk [50], [51], [52], while other studies did not show that low-level consumption of alcohol increases breast cancer risk in premenopausal women [53].

The effect of alcohol drinking among nulliparous women was significantly elevated compared to that among parous women. This result is consistent with prior studies which suggests that the carcinogenic effect of alcohol consumption is strongest between menarche and the first full term pregnancy and could be implicated in both increased risk of breast cancer and benign breast disease [54], [55], [56]. Starting to drink at a young reproductive age (<30 year, particularly during 25–29 years) was associated with an increased risk of breast cancer in our study sample. This finding is consistent with other studies [43], [57], [58], [59].

In addition, alcohol drinking among women with BMI >25 kg/m^2^, clinically defined as overweight or obese, had a greater risk of developing breast cancer. This suggests that obesity and alcohol drinking may have joint effects on risk of breast cancer among women. Being overweight or obese has been shown to be a risk factor for postmenopausal breast cancer [60], [61]. The proposed mechanism in both animal models and humans of the mediating effect of alcohol as well as adiposity's effect on serum estradiol and insulin levels may account for the interaction effect observed between BMI and alcohol consumption [62], [63].

Heterogeneity in the level of alcohol consumption was observed among women in three African countries. Using a definition of alcohol drinking as having at least one drink every week for a continuous 6 months or more, the percentage of ever drinkers in Nigeria, Cameroon, and Uganda were 5.0%, 34.6%, and 50.0%, respectively. This is in line with previous surveys of alcohol consumption in the three countries, although it is not straightforward to directly compare with previous studies due to different definition of alcohol drinking. According to the WHO country surveys conducted from 2003 to 2005, the percent of women who are lifetime abstainers in Nigeria, Cameroon, and Uganda were 50.9%, 39.8%, 36.7%, respectively [64], [65], [66]. Heterogeneity in the relationship between alcohol drinking and breast cancer was also observed across the three countries. In Nigeria and Cameroon, women who drank alcohol had a moderate increased risk of breast cancer compared to never-drinkers, whereas in Uganda there was only a marginal increase in risk. A small sample size (n = 380) may explain for these non-significant findings in Uganda. A previous study found that a larger proportion of Nigerian women who drank alcohol regularly tended to be heavy drinkers compared to Ugandan women [29], which may also account for the weak correlation between alcohol consumption and breast cancer risk for women in Uganda. Larger sample size in Uganda and Cameroon are desirable to confirm these study findings.

Although some studies have suggested that recall bias only had minor effects on alcohol reporting and on the estimation of risk for breast cancer [67], we could not exclude the possibility of recall bias in reporting alcohol consumption in our study. Due to the retrospective nature of the study, participants might have difficulty in recalling alcohol consumption patterns, especially if the patterns changed over time for older women. In addition, cultural stigmas against alcohol use among women might also have resulted in under-reporting of alcohol consumption [68], [69], [70]. Nigerian women might tend to under-report alcohol consumption, as it is often considered culturally inappropriate for them to drink alcohol outside of religious settings [70]. The majority of subjects in the study were considered to be light drinkers whereas there were few moderate and heavy drinkers. This observation could also account for the lack of a dose-response relationship with respect to the amount of alcohol consumed. Our study did not assess alcohol consumption at each stage of a woman's life, and therefore, it was assumed that alcohol consumption was constant over a subject's lifetime. Since ethanol and its primary metabolite, acetaldehyde, can affect estrogen levels and are involved in carcinogenic pathways [71], alcohol consumption later in life may further accelerate genetic mutations.

In conclusion, our study found a positive relationship between alcohol consumption and breast cancer risk among sub-Saharan African women. The adoption of a Western lifestyle and erosion of many traditional customs that prohibited alcohol drinking has led to drastic changes in alcohol consumption patterns, including greater drinking among young people and women [72], [73], [74]. According to the 2011 WHO data, 25.3% of the population in Africa is displaying an increase in five-year trends for recorded adult per capita alcohol consumption [75]. Surveys from 2005 also show that in Nigeria and Uganda, total adult (defined as 15 years or older) per capita alcohol consumption (10.00–12.49 liters) has already exceeded the per capita alcohol consumption in the United States (7.50–9.99) [75]. If we assumed a common odds ratio of 1.62 and observed alcohol prevalence in the three countries, 4.6%, 19.5%, and 20.1% of breast cancer cases can be attributed to alcohol drinking in Nigeria, Cameroon, and Uganda, respectively. As breast cancer incidence is increasing in African countries and alcohol drinking is increasingly popular among African women [29], [30], [31], [32], [46], [47], public health programs in Africa should take proactive measures to prevent breast cancer through addressing the modifiable risk factor of alcohol consumption, including providing current information on the risk of alcohol consumption among women and advocating proper regulation of alcohol sale through more effective alcohol policies [76], [77], [78].

## Supporting Information

Table S1 **Multivariable logistic regression analyses of alcohol consumption and breast cancer risk among women in Nigeria, Cameroon and Uganda, 1998–2013: Complete case analysis (N = 4157).**
(DOC)
