# Supplementary material for: Alcohol Consumption and Breast Cancer Risk among Women in Three Sub-Saharan African Countries
Source: PLoS One. 2014 Sep 8;9(9):e106908. doi: 10.1371/journal.pone.0106908 (PMC4157846; doi:10.1371/journal.pone.0106908)
Supplement: Table S1 — Multivariable logistic regression analyses of alcohol consumption and breast cancer risk among women in Nigeria, Cameroon and Uganda, 1998–2013: Complete case analysis (N = 4157). (DOC) [file pone.0106908.s001.doc]

**Supplementary Information**

**Table S1.** Multivariable logistic regression analyses of alcohol consumption and breast cancer risk among women in Nigeria, Cameroon and Uganda, 1998-2013: Complete case analysis (N=4157)

| **Variable** | **Nigeria** | | **Cameroon** | **Uganda** | **Overall** |
| --- | --- | --- | --- | --- | --- |
|  | **Adjusted OR**  **(95% CI)^†^** | | **Adjusted OR**  **(95% CI)^†^** | **Adjusted OR**  **(95% CI)^†^** | **Adjusted OR**  **(95% CI)**^‡^ |
| **Ever drank alcohol** | |  |  |  |  |
| **No** | 1.0 (ref.) | | 1.0 (ref.) | 1.0 (ref.) | 1.0 (ref.) |
| **Yes** | 1.78 (1.30 – 2.44) | | 1.42 (0.82 – 2.47) | 0.90 (0.44 – 1.83) | 1.64 (1.29 – 2.07) |
|  |  | |  |  |  |
| **Status of drinking** |  | |  |  |  |
| **Never** | 1.0 (ref.) | | 1.0 (ref.) | 1.0 (ref.) | 1.0 (ref.) |
| **Past** | 1.76 (1.16 – 2.69) | | 1.12 (0.47 – 2.68) | 1.41 (0.62 – 3.21) | 1.70 (1.24 – 2.35) |
| **Current** | 1.69 (1.04 – 2.76) | | 1.51 (0.82 – 2.76) | 0.50 (0.21 – 1.22) | 1.51 (1.10 – 2.07) |
|  |  | |  |  |  |
| **Age at first drink, year** |  | |  |  |  |
| **Never drank** | 1.0 (ref.) | | 1.0 (ref.) | 1.0 (ref.) | 1.0 (ref.) |
| **≤18** | 1.67 (0.74 – 3.78) | | 0.78(0.27 – 2.26) | 1.19 (0.46 – 3.08) | 1.67 (1.04 – 2.69) |
| **19-24** | 1.73 (0.94 – 3.15) | | 1.45 (0.69 – 3.19) | 0.84 (0.26 – 2.74) | 1.94 (1.23 – 3.05) |
| **25-29** | 1.90 (1.08 – 3.35) | | 3.30 (1.22 – 8.94) | 0.65 (0.14 – 3.03) | 1.97 (1.33 – 2.90) |
| **≥30** | 1.74 (0.99 – 3.05) | | 1.07 (0.45 – 2.53) | 0.67 (0.26 – 1.75) | 1.18 (0.80 – 1.73) |
| ***P*-value for trend** | <0.001 | | 0.17 | 0.43 | 0.001 |
|  |  | |  |  |  |
| **Duration of alcohol drinking, year** | | |  |  |  |
| **Never**  **drank** | 1.0 (ref.) | | 1.0 (ref.) | 1.0 (ref.) | 1.0 (ref.) |
| **1-9** | 1.39 (0.87 – 2.21) | | 1.62 (0.56 – 4.72) | 1.15 (0.43 – 3.11) | 1.66 (1.15 – 2.40) |
| **10-19** | 1.54 (0.87 – 2.54) | | 1.71 (0.72 – 4.06) | 1.63 (0.49 – 5.41) | 1.71 (1.15 – 2.55) |
| **≥ 20** | 2.72 (1.38 – 5.38) | | 2.33 (1.08 – 5.00) | 0.79 (0.27 – 2.35) | 1.90 (1.27 – 2.82) |
| ***P*-value for trend** | <0.001 | | 0.05 | 0.86 | <0.001 |
| **Per 10-year increase** | 1.27 (1.00 – 1.61) | | 1.47 (1.19 – 1.82) | 0.99 (0.69 – 1.40) | 1.26 (1.11 – 1.43) |
|  |  | |  |  |  |
| **Average amount of alcohol**  **consumed daily, gram** | | |  |  |  |
| **Never drank** | 1.0 (ref.) | | 1.0 (ref.) | 1.0 (ref.) | 1.0 (ref.) |
| **0.1–4.9** | 2.86 (1.53 – 5.36) | | 1.18 (0.52 – 2.67) | 1.39 (0.39 – 4.91) | 1.95 (1.31 – 2.92) |
| **5.0–9.9** | 1.87 (1.15 – 3.04) | | 0.32 (0.13 – 0.79) | 0.31 (0.06 – 1.56) | 1.34 (0.92 – 1.95) |
| **≥10** | 1.07 (0.63 – 1.81) | | 0.45 (0.19 – 1.08) | 1.06 (0.12 – 9.48) | 0.99 (0.65 – 1.50) |
| ***P*-value for trend** | 0.17 | | 0.01 | 0.44 | 0.82 |
| **Per 10g increase** | 1.40 (0.87 – 2.25) | | 0.35 (0.15 – 0.80) | 0.47 (0.07 – 3.17) | 1.05 (0.72 – 1.53) |
|  |  | |  |  |  |
| **Duration and amount of alcohol consumed, gram-years** |  | |  |  |  |
| **Never**  **drank** | 1.0 (ref.) | | 1.0 (ref.) | 1.0 (ref.) | 1.0 (ref.) |
| **0.1–29.9** | 1.26 (0.75 – 2.14) | | 0.93 (0.21 – 4.21) | 2.48 (0.28 – 22.1) | 1.33 (0.84 – 2.11) |
| **30.0–79.9** | 2.82 (1.51 – 5.27) | | 1.44 (0.55 – 3.76) | 0.32 (0.06 – 1.77) | 2.03 (1.28 – 3.20) |
| **80.0–159.9** | 1.79 (0.91 – 3.51) | | 1.04 (0.40 – 2.73) | 0.78 (0.18 – 3.39) | 1.57 (0.97– 2.52) |
| **≥160.0** | 1.28 (0.65 – 2.54) | | 0.92 (0.41 – 2.06) | 0.98 (0.14 – 6.97) | 1.14 (0.71 – 1.83) |
| ***P*-value for trend** | 0.77 | | 0.47 | 0.59 | 0.95 |
| **Per 10 gram-year increase** | 1.00 (0.99 – 1.01) | | 1.01 (0.99 – 1.02) | 0.98 (0.91 – 1.05) | 1.00 (0.99 – 1.01) |
|  |  | |  |  |  |
| **Type of alcohol ^۩^** |  | |  |  |  |
| **Never drank** | 1.0 (ref.) | | 1.0 (ref.) | 1.0 (ref.) | 1.0 (ref.) |
| **Beer** | 1.79 (1.35 – 2.38) | | 1.34 (0.80 – 2.27) | 0.69 (0.41 – 1.16) | 1.43 (1.16 –1.77) |
| **Wine** | 2.28 (1.18 – 4.39) | | 0.71 (0.34 – 1.50) | 1.02 (0.57 – 1.84) | 1.17 (0.83 –1.64) |
| **Spirits** | 1.69 (0.85 – 3.34) | | 1.02 (0.42 – 2.47) | 1.40 (0.77 – 2.54) | 1.39 (0.96 – 2.02) |

^†^ Adjusted for age at diagnosis or interview (categorical), ethnicity, education (categorical), age at menarche (categorical), number of live births (categorical), age at first live birth (categorical), menopausal status, family history of breast cancer, benign breast disease, hormonal contraceptive use, BMI (continuous), and height (continuous);

^‡^ Adjusted for the above variables and study site;

^۩^ Includes drinkers who drink a combination of alcoholic beverages
